# Accurate classification of COVID‐19 patients with different severity via machine learning

**DOI:** 10.1002/ctm2.323

**Published:** 2021-02-26

**Authors:** Chaoyang Sun, Yong Bai, Dongsheng Chen, Liang He, Jiacheng Zhu, Xiangning Ding, Lihua Luo, Yan Ren, Hui Xing, Xin Jin, Gang Chen

**Affiliations:** ^1^ Cancer Biology Research Center (Key Laboratory of the Ministry of Education) Tongji Medical College Tongji Hospital Huazhong University of Science and Technology Wuhan China; ^2^ Department of Gynecologic Oncology Tongji Hospital Tongji Medical College Huazhong University of Science and Technology Wuhan China; ^3^ BGI‐Shenzhen Shenzhen China; ^4^ BGI Education Center University of Chinese Academy of Sciences Shenzhen China; ^5^ Department of Obstetrics and Gynecology Xiangyang Central Hospital Hubei University of Arts and Science Xiangyang China; ^6^ School of Medicine South China University of Technology Guangzhou China; ^7^ Guangdong Provincial Key Laboratory of Human Disease Genomics Shenzhen Key Laboratory of Genomics BGI‐Shenzhen Shenzhen China

Dear Editor,

Infection of severe acute respiratory syndrome coronavirus 2 (SARS‐CoV‐2) could cause dramatic response in coronavirus disease 2019 (COVID‐19) patients at multi‐omics level,^1^, ^2^, ^3^ thus it is essential to systematically assess the pathogenesis of COVID‐19. In our previous study, we presented the first trans‐omics landscape of 236 COVID‐19 patients with 4 clinical severity groups (including asymptomatic, mild, severe and critically ill cases) and found that the mild and severe COVID‐19 patients shared several similar characteristics.^4^ However, it is crucial to discriminate mild from severe COVID‐19 patients to prevent the latter from the progression of disease by facilitating early intervention. Herein, we developed an extreme gradient boosting (XGBoost) machine‐learning model to predict the COVID‐19 severities by leveraging multi‐omics data. Briefly, we randomly stratified samples for the training set (80%) and the independent testing set (20%) (Figure 1A, see Methods in the Supporting Information). After normalization, a total of 297 multi‐omics features were preliminarily selected by applying a hybrid method (see Methods in the Supporting Information). The XGBoost model was trained on the training set with the preliminarily selected features, achieving mean micro‐average AUROC (area under the receiver operating characteristic curve) and mean micro‐average AUPR (area under the precision‐recall curve) of 0.9715 (95% CI, 0.9497–0.9932) and 0.9495 (95% CI, 0.9086–0.9904), respectively (Figure 1B and C). This showed strong generalizable discrimination among the four severities based on fivefold cross‐validation over 100 iterations.

**FIGURE 1:**
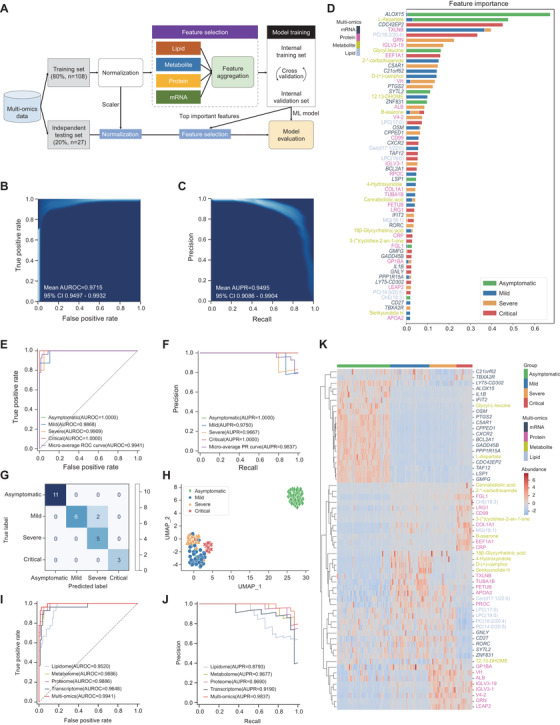
Identification of biomarkers associated with COVID‐19 severity using machine‐learning model. (A) Flowchart of developing XGBoost machine‐learning model. The model was trained with cross‐validation using a training set (*n* = 108) after normalization and feature selection and re‐trained with the identified top 60 important features. The re‐trained model was further applied to assess generalization and performance using the independent testing set (*n* = 27). (B and C) Performance of the model learned in the training set in terms of mean micro‐average AUROC (B) and mean micro‐average AUPR (C). Rasterized density plot of ROC (B) and PR (C) curve data from fivefold cross‐validation for 100 iterations. (D) Top 60 important features (mRNA *n* = 23; proteins *n* = 19; metabolites *n* = 11; lipids *n* = 7) ranked by SHAP value. The stacked bar indicates the average impact of the feature on the model output magnitude for different classes. (E and F) Performance of XGBoost model based on the top 60 features for distinguishing the four groups of COVID‐19 patients' severity in an independent testing set in terms of AUROC (E) and A UPR (F). (G) Confusion matrix for predicting COVID‐19 severity in the independent testing set (*n* = 27). (H) UMAP plot based on the top 60 features showing the distinct separation among the four types of COVID‐19 severities in the whole data set (patients *n* = 135). (I and J) Comparison of performance of models learned between each single‐omics data with that of multi‐omics data in an independent testing set in terms of AUROC (I) and AUPR (J). (K) Heatmap demonstrating the top 60 features profiles of the four groups of COVID‐19 patients severity in the whole dataset (patients *n* = 135)

The multi‐omics features were prioritized and ranked by the XGBoost model and the SHAP (SHapley Additive exPlanations, see Methods in the Supporting Information) value. Top 60 important features were further selected consisting of 19 proteins, 11 metabolites, 7 lipids, and 23 mRNAs (Figure 1D, Figures S1‐S4). With the top 60 features, the XGBoost model was re‐trained and validated, resulting in a micro‐average AUROC and micro‐average AUPR of 0.9941 and 0.9837 based on an independent testing set, respectively (Figure 1E and F). The confusion matrix (Figure 1G) showed that all patients in the independent testing set were correctly identified, except for two mild patients who were predicted as severe. For further validation, we trained different XGBoost models through the same training protocol with each single‐omics data. Results demonstrated that the XGBoost model outperformed models trained using single‐omics features (Figure 1I and J, Figure S5). Furthermore, we trained an additional XGBoost model based on the 24 features identified in Guo's method^5^ (two proteins and three metabolites were not detected in our experiment), leading to micro‐average AUROC and micro‐average AUPR in independent testing set be 0.9305 and 0.8300, respectively (Figure S5), which may be partially due to the different purposes for model construction. Guo's method sought to distinguish severe patients from nonsevere patients, whereas we attempted to identify four groups of COVID‐19 patients' severity. The uniform manifold approximation and projection (UMAP) plot showed distinct separation of the four severity groups(Figure 1H). Furthermore, we calibrated our model using Platt scaling method in a one‐versus‐rest fashion. The expected calibrator error (ECE) and brier score (BS) were computed to evaluate calibration (see Methods in the Supporting Information). As a result, the ECE for the uncalibrated model and the calibrated model was 0.0773 and 0.0996, respectively, whereas the BS for the uncalibrated model and the calibrated model was 0.0312 and 0.0353, respectively (Figure S6). These results suggested that the output probabilities of our model can represent uncertainty about prediction. Together, our results implied that the XGBoost model based on the top 60 multi‐omics features could precisely differentiate COVID‐19 patient severity status.

Many machine learning‐based models have been developed to predict outcomes of patients with COVID‐19. Nevertheless, most of those models were created based on computed tomography images or several diagnostic predictors such as age, body temperature, clinical signs and symptoms, complications, epidemiological contact history, pneumonia signs, neutrophils, lymphocytes, and C‐reactive protein (CRP) levels. Recently, COVID‐19 patients that may become severe were identified by applying a prediction model that was developed using proteomic and metabolomic measurements.^5^ Although all these models reported promising predictive performance with high C‐indices, they carried a high risk of bias according to the Prediction model Risk Of Bias ASsessment Tool (PROBAST).^6^ This was because most prediction models did not exclude the patients with severe comorbidities and had a high risk of bias for the participant group or used nonrepresentative controls, making the prediction results unreliable. Here, we minimized the selection bias using strict inclusion and exclusion criteria (see Methods in the Supporting Information). According to the results of the prediction model, most mRNAs were highly correlated with asymptomatic patients (Figure 1K; Figure S1). Multi‐omics features such as *TBXA2R*, *ALOX15*, *IL1B*, *IFIT2*, *BCL2A1*, *LSP1*, glycyl‐L‐leucine, and l‐aspartate were highly expressed in the asymptomatic group, and thus may potentially yield crucial diagnostic biomarkers for identifying asymptomatic COVID‐19 patients. In the critical illness group, besides CRP which has already been used to monitor the severity of COVID‐19, some immune‐related features, such as *EEF1A1*, *FGL1*, *LRG1*, *CD99*, *COL1A1*, cholinesterase(18:3), monoacylglyceride(18:1), cannabidiolic acid, and beta‐asarone were found to be highly expressed in the critical group. Moreover, two transcription factor encoding genes, *ZNF831* and *RORC*, closely associated with immune response were lowly expressed in critical patients. Using these features, we could optimize existing approaches to improve the accuracy and sensitivity of detection based on nucleic acid testing and predict asymptomatic patient prognosis more accurately. With the assistance of this machine‐learning model, we could help identify individuals with a high risk of poor prognosis in advance, and prevent progression in time to minimize individual, medical, and social costs.

In summary, we developed an XGBoost‐based model by integrating multi‐omics data to dissect subtle changes in gene expression and pathways of COVID‐19 patients with different severity levels. Our model reached micro‐average AUROC and micro‐average AUPR as high as 0.9941 and 0.9837, respectively, which could clearly distinguish patients from different severity groups and accurately predict pathological status. In addition, analysis of the top 60 multi‐omics features demonstrated that our model had the potential of discovering molecules associated with the pathogenesis of COVID‐19. Overall, the methodology employed in this study could be widely applied for the study of other diseases and provide clues for the control and treatment of patients suffering from COVID‐19 and many other infectious diseases.

## CONFLICT OF INTEREST

The authors declare no conflict of interest.

## AUTHOR CONTRIBUTIONS

Gang Chen, Xin Jin, Yong Bai, Peng Wu, and Yan Ren contributed to project design. Yong Bai developed the machine learning model. Chaoyang Sun, Yong Bai, Dongsheng Chen, Jiacheng Zhu, Xiangning Ding, and Lihua Luo contributed to data interpretation and visualization. All authors contributed to writing the original draft.

## Supporting information

Supporting information
