# Supplementary material for: Accurate classification of COVID‐19 patients with different severity via machine learning
Source: Clin Transl Med. 2021 Feb 26;11(3):e323. doi: 10.1002/ctm2.323 (PMC7908044; doi:10.1002/ctm2.323)
Supplement: Supplementary file 1 — Supporting information [file CTM2-11-e323-s001.docx]

# Accurate classification of COVID-19 patients with different severity using machine learning

**SUPPLEMENTARY MATERIALS**

**Methods**

**Patients Enrollment and Sample Preparation**

The detailed inclusion and exclusion criteria were as following, which was also available in our previous paper^1^. Briefly, patients were diagnosed following the guidelines for COVID-19 diagnosis and treatment (Trial Version 7) released by the National Health Commission of the People’s Republic of China. Patients with any of the following comorbidities were excluded from this study: surgical history, hypertension, immunodeficiency disease, chronic hepatitis, tuberculosis, coronary heart disease, diabetes, chronic kidney disease, cerebrovascular disease, chronic obstructive pulmonary disease, malignancy. Subsequently, patients were classified into four groups (critical, severe, mild, and asymptomatic) according to their disease severity. The critically ill patients fulfilled the definition of at least one of the following conditions: (1) acute respiratory distress syndrome (ARDS) requiring mechanical ventilation, (2) other organ failure requiring ICU admission (3) shock. Severe patients met at least one of the following criteria : (1) oxygen saturation ≤93% at resting state, (2) respiratory rate ≥ 30 times/min, (3) arterial partial pressure of oxygen (PaO2)/fraction of inspired oxygen (FiO2) ≤300 mmHg, (4) pulmonary imaging examination showed that the lesions significantly progressed by more than 50% within 24-48 hours. Mild patients were defined as having fever, respiratory symptoms, or imaging evidenced pneumonia. The patients without fever and any respiratory symptoms were defined as asymptomatic.

### Data Preprocessing for Machine Learning

Extreme gradient boosting (XGBoost)^2^, an ensemble algorithm of decision trees, was developed to predict patient severity status based on multi-omics data of mRNA transcripts (n=13323, mRNAs with FPKM >1 in at least one sample were retained), proteins (n=634), metabolites (n=814), lipids (n=742) from 135 patients (asymptomatic n=53, mild n=39, severe n=27, and critical n=16) using the open-sourced Python package (<https://xgboost.readthedocs.io/en/latest/>, version=1.0.0). Features with missing values were filled with the smallest value of that feature among all the samples. We employed random stratified sampling to select 108 patients (80% of patient cohort) as the training set (asymptomatic n = 42, mild n = 31, severe n = 22, and critical n = 13), while the remaining 27 patients were used as the independent testing set (**Figure 1A)**. A fixed random number seed was used to ensure reproducibility of the results. The multi-omics data in training set was first normalized by centering and scaling for each sample to have mean zero and unit standard deviation. The estimated mean value and standard deviation for each feature from the training set were applied to the corresponding features in the testing phase afterwards.

### Feature Selection

Due to high dimensional multi-omics data and thus may decrease model’s performance if irrelevant features were included, we proposed a hybrid feature selection method to remove redundant and noise features. In this method, both mutual information (MI)-based technique and Boruta^3^ algorithm were employed to obtain relevant subset of raw features. The MI-based technique was one of filter methods to select relevant features. It calculated weight by taking into account the relationship between features based on mutual information, and assigned the weight to each feature based on degree of relevance of features to class labels. We then selected 30% of features with the highest weights (Scikit-learn, version=0.23.1). The Boruta algorithm was one of wrapper methods to select subset of features based on a random forest machine learning algorithm that was used to measure feature importance. One feature was selected by Boruta only if its importance was greater than a threshold that was defined as the highest feature importance recorded among shadow features. The shadow features were obtained by permuting a copy of the real features across samples to destroy the relationship with the outcome. In Boruta, we applied random forest classifier with default parameters from Scikit-learn library. Due to imbalanced training set for each group of COVID-19 patients, we incorporated different weights of classes into the random forest classifier. The weight of class $c$ in the training dataset was calculated as $w_{c}=\frac{N}{kN_{c}}$, where $N$ is the total number of samples, $k$ is the number of unique classes, $N_{c}$ is the number of samples from class $c$.Python library BorutaPy (<https://github.com/scikit-learn-contrib/boruta_py>) was used to conduct Boruta algorithm using default parameters and a fixed random number seed. The final subset of relevant features was determined by computing intersection of subset features resulting from MI-based technique and Boruta algorithm. This procedure was repeated for each single-omics data and the final subset of relevant features was aggregated together for each sample.

### Model Training and Top Important Feature Identification

We performed a basic grid search algorithm with 5-fold cross validation to optimize XGBoost parameters while maximizing weighted F1 score because of the imbalanced training set (that is, the various number of samples in different patient group of COVID-19 severity). We employed the same approach as that used in the Boruta algorithm to calculate weight for each class and then assigned each sample with its corresponding class weight during the model training phase. This calculation led to higher weights for minority classes while lower weights for majority classes, and therefore can alleviate the issue of class imbalance in the training dataset. We used softmax as learning objective function due to the multi-class identification problem in this study. For a given sample, the softmax function produced a vector of probabilities for each of classes. As a common practice in machine learning community, the maximum value and its corresponding index of the vector were chosen as the sample’s predicted probability and class label, respectively. To evaluate overall performance of our model during the model training phase, we performed 5-fold cross validation to calculate metrics of mean micro-average ROC curve with AUROC value and a mean micro-average PR curve with AUPR value in a one-vs-rest fashion. This procedure was repeated 100 iterations. Consequently, the favorable values for the tuned XGBoost parameters were identified as follows: the maximum depth of trees was 8, number of decision trees was 55, minimum sum of instance weight needed in a child of a tree was 1, partitioning-leaf-node parameter was 0.4, subsample ratios of training instances for constructing each tree was 0.7, subsample ratios of columns was 0.9, learning rate was 0.05 and L1 regularization parameter was 0.005. After obtaining the favorable parameter values, the XGBoost model was trained using the entire training set.

We applied the SHAP (SHapley Additive exPlanations)^4,5^ approach to measure feature importance for the XGBoost model. SHAP was a unified method to explain machine learning prediction based on game theoretically optimal Shapley values. To explain the prediction of a sample by the ML model, SHAP computed the contribution of each feature to the prediction, which was quantified using Shapley values from coalitional game theory. The Shapley value was represented as an additive feature attribution method, providing the average of the marginal contributions across all permutations of features and distribution of model prediction among features. As an alternative to permutation feature importance, SHAP feature importance was based on magnitude of feature attributions. The absolute Shapley values per feature across the data was further averaged as the global importance was needed. We ranked the features importance in descending order and picked the top 60 most important features. The stacked bar indicated the average impact of the feature on model output magnitude for different classes. We used the Python library to implement the SHAP algorithm (<https://github.com/slundberg/shap>). We re-trained the final XGBoost model based on the top 60 important features with the favorable model parameters using the entire training set.

**Machine Learning Model Evaluation**

We evaluated the performance of the final XGBoost model as follows. We first normalized multi-omics data from the unseen 20% independent testing set using the mean value and standard deviation obtained during the training phase. Subsequently, features were screened based on the top 60 important features, followed by classification process using the final XGBoost model. The performance metrics included ROC curves with AUROC values, PR curves with AUPR values for each class, while micro-average ROC curves with AUROC values and micro-average PR curves with AUPR values for overall. In addition, confusion matrices (predicted label as the index of maximum value of the predicted probability vector) and UMAP plots (with parameters of the number of neighbors being 10, the minimum distance between points being 0.5 and the distance metric being Manhattan) were also generated for evaluating the performance.

To compare the performance of model based on multi-omics data to that based on single-omics data, we trained XGBoost model for single-omics data using the same training protocol as multi-omics data, except that we only empirically picked top 30 important features to train the final single-omics based XGBoost model. Moreover, we selected 20 proteins and 4 metabolites mentioned in Guo’s method^6^, where 2 proteins and 1 metabolite were not found in our data set and additional 2 metabolites were excluded from our analysis due to their identification with lower confidence. We trained XGBoost model using these 24 features with the same training protocol. Those models were evaluated on the unseen 20% independent testing set and calculated the same the performance matrices as mentioned above (**Figure S5**). To further investigate the top 60 important features, we applied Mann-Whitney U-test (multiple comparisons correction with Bonferroni) to test statistically significant difference of each normalized features between severity groups (**Figure S1-S4**).

**Model Calibration**

To verify consistency between the predicted probabilities and the empirical frequencies observed from actual outcomes, we applied Platt scaling method (doi: http://citeseer.ist.psu.edu/viewdoc/summary?doi=10.1.1.41.1639) to calibrate our classification model. The Platt scaling method converted probabilistic predictions to posterior probabilities over classes by passing them through a sigmoid (called calibrator):

$$p\left( y_{i}=1 | f_{i} \right)=\frac{1}{1+\exp\left( Af_{i}+B \right)}$$

where $y_{i}\in\left\{ 0, 1 \right\}$ and $f_{i}\in\left[ 0, 1 \right]$ were true class label and predicted probability of the uncalibrated model for sample $i$, respectively, $A$ and $B$ were two paramters learned by maximum likelihood estimation. We trained the calibrator using 5-fold cross validation approach to obtain unbiased predictions. Due to the multi-class identification problem in this study, we calibrated for each class separately in a one-vs-rest fashion.

We used expected calibrator error (ECE)^7,8^ and brier score (BS) to evaluate how well the model calibrated. ECE was a commonly used summary statistic of calibration that measures the difference between the expected probability and fraction of positives.

Let $M$ be the number of equally-spaced bins, $B_{m}$ the set of samples whose predicted probability falled into the interval $I_{m} =(\left. \frac{m-1}{M}, \frac{m}{M} \right]$. The fraction of positives for $B_{m}$ can be computed by:

$$\text{acc}\left( B_{m} \right)=\frac{1}{\left| B_{m} \right|} \sum_{i\in B_{m}} I\left( \hat{y}_{i}=y_{i} \right)$$

where $\hat{y}_{i}$ and $y_{i}$ were predicted and true class label for sample $i$. On the other hand, the expected probability of $B_{m}$ was defined as:

$$\text{conf}\left( B_{m} \right)= \frac{1}{\left| B_{m} \right|}\sum_{i\in B_{m}} \hat{p_{i}}$$

where $\hat{p}_{i}$ was the predicted probability for sample $i$. ECE can then be approximated as follows:

$$\text{ECE} = \sum_{m=1}^{M} \frac{\left| B_{m} \right|}{n}\left| \text{acc}\left( B_{m} \right)-\text{conf}\left( B_{m} \right) \right|$$

where $n$ is the total number of samples. We chose $M=10$ to compute ECE.

In addition to ECE, brier score (BS) was used to measure the mean squared difference between the predicted probability and the actual outcome. The brier score was calculated by:

$$\text{BS} = \frac{1}{n}\sum_{i=1}^{n} {(y_{i}-\hat{p_{i}})}^{2}$$

**Data Visualization**

All the figures in our manuscript were plotted using software packages of Seaborn (version 0.9.0) and Matplotlib (version 3.2.2).

**Data and source code availability**

The data that support the findings of this study, including the genome-wide association test summary statistics, expression matrices for multi-omics have been deposited in CNSA (China National GeneBank Sequence Archive) in Shenzhen, China with accession number CNP0001126 [http://ftp.cngb.org/pub/CNSA/data3/CNP0001126/Annotation_stats/].Custom scripts for data analysis in this study were present in https://github.com/y-bai/multiomics-covid19/.

**References**

1. Wu P, Chen D, Ding W, et al. The Trans-omics Landscape of COVID-19. *medRxiv*. Published online July 22, 2020:2020.07.17.20155150.

2. Chen T, Guestrin C. XGBoost: A scalable tree boosting system. In: *Proceedings of the ACM SIGKDD International Conference on Knowledge Discovery and Data Mining*. Association for Computing Machinery; 2016:785-794.

3. Kursa MB, Rudnicki WR. Feature Selection with the Boruta Package. *J Stat Softw*. 2010;36(11).

4. Lundberg SM, Lee SI. A unified approach to interpreting model predictions. In: *Advances in Neural Information Processing Systems*. ; 2017:4765-4774.

5. Lundberg SM, Erion G, Chen H, et al. From local explanations to global understanding with explainable AI for trees. *Nat Mach Intell*. 2020;2(1):56-67. h

6. Shen B, Yi X, Sun Y, et al. Proteomic and Metabolomic Characterization of COVID-19 Patient Sera. *Cell*. 2020;182(1):59-72.e15.

7. Guo C, Pleiss G, Sun Y, Weinberger KQ. On calibration of modern neural networks. *Proc Int Conf Mach Learn*. Published online 2017:1321–1330.

8. Naeini MP, Cooper G, Hauskrecht M. Obtaining well calibrated probabilities using bayesian binning. In: *Proceedings of the AAAI Conference on Artificial Intelligence*. Vol 29. ; 2015.

**SUPPLEMENTARY FIGURES**

**
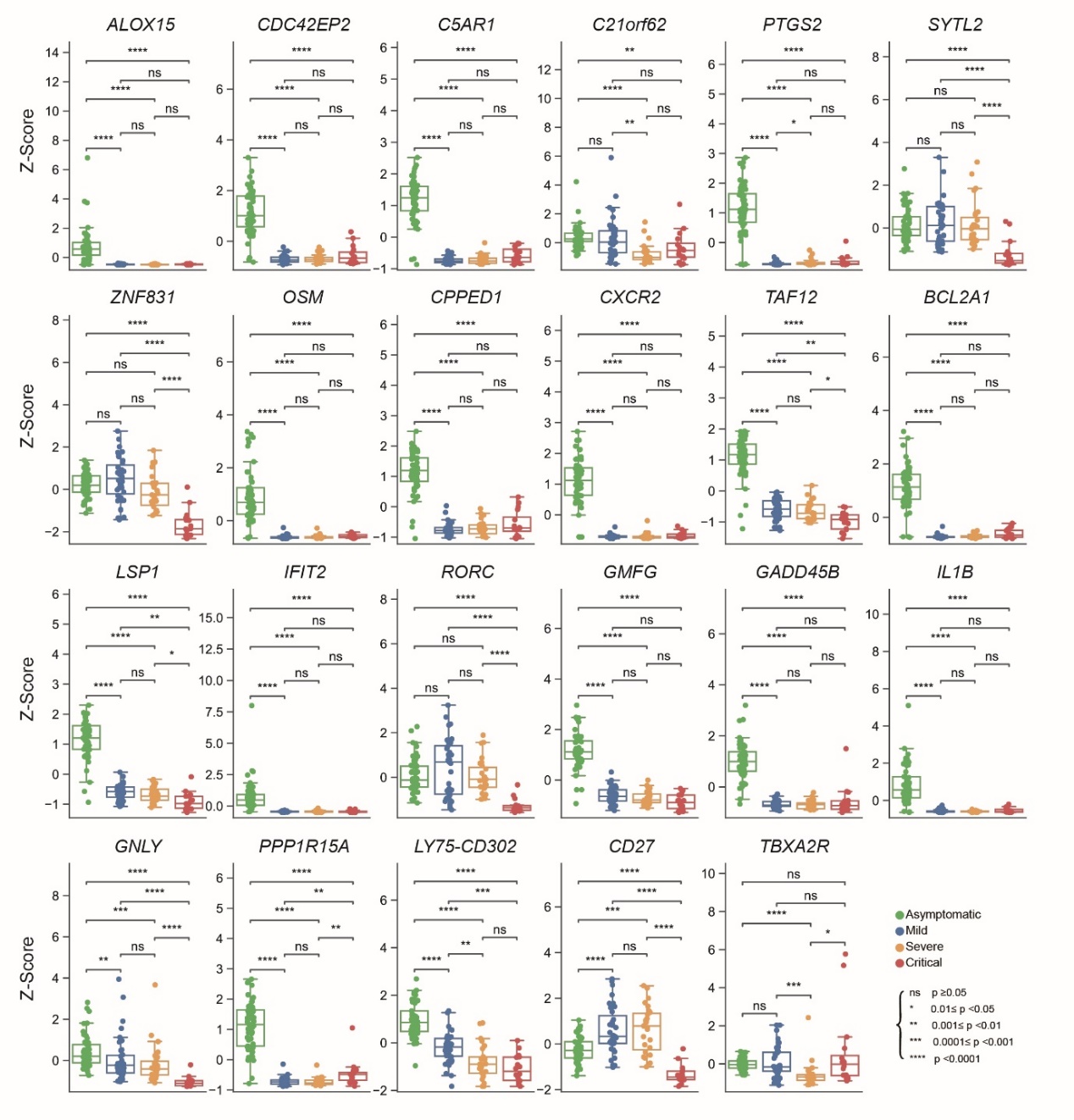
**

**Figure S1. Distribution of mRNAs from the Top 60 Important Multi-omics Features among the 4 Types of COVID-19 Severities**

**
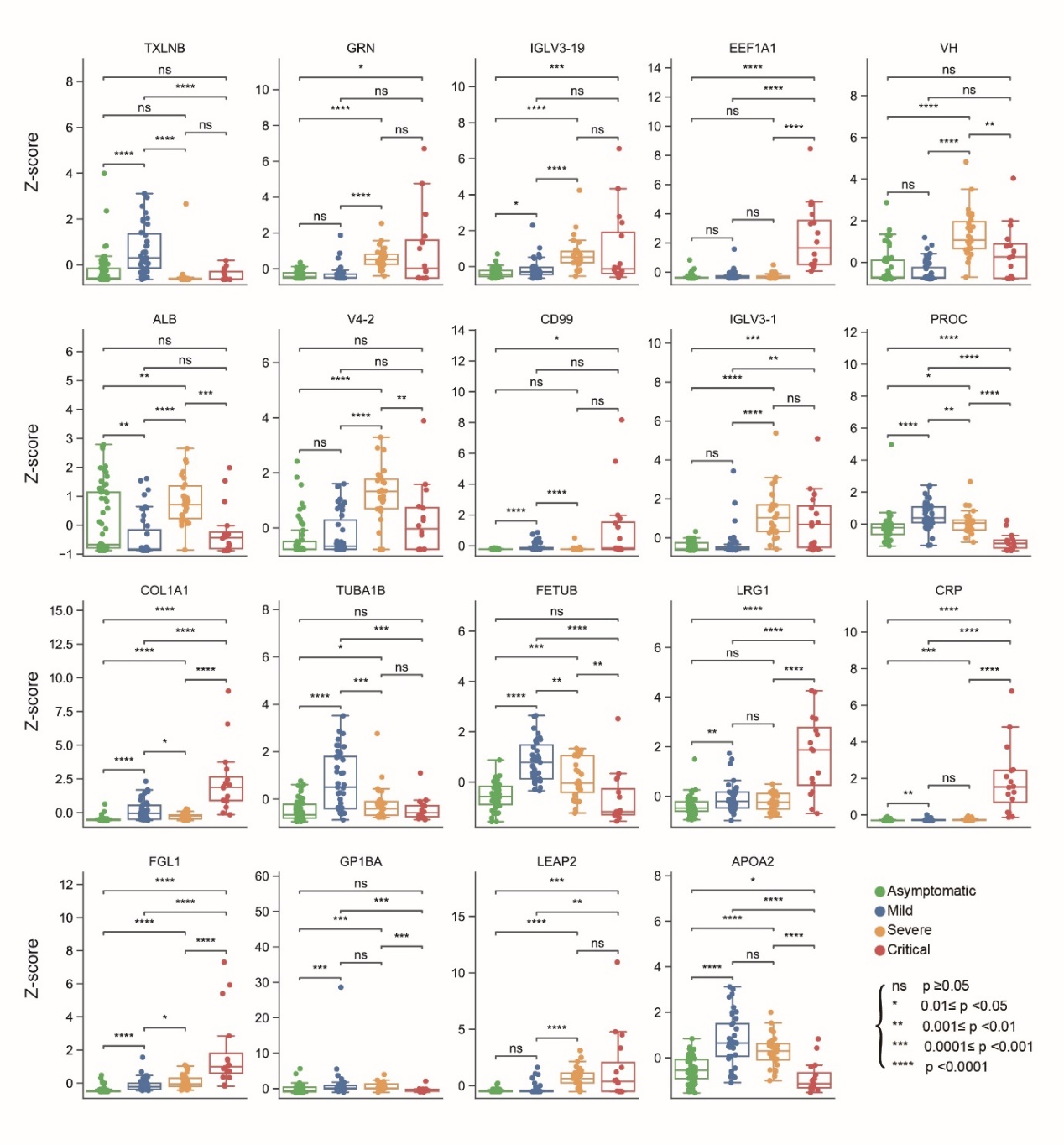
**

**Figure S2. Distribution of Proteins from the Top 60 Important Multi-omics Features among the 4 Types of COVID-19 Severities**

**
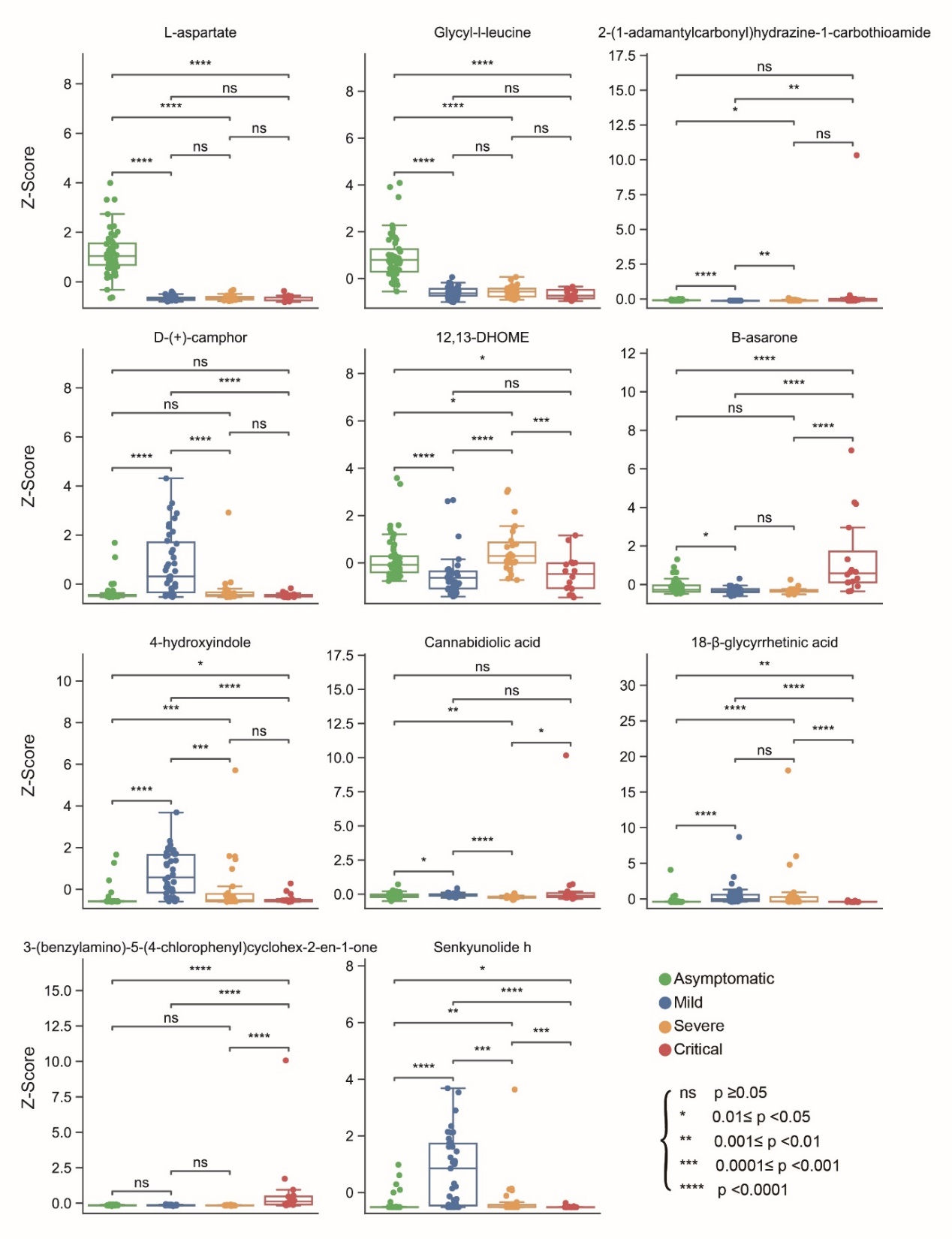
**

**Figure S3. Distribution of Metabolites from the Top 60 Important Multi-omics Features Among the 4 Types of COVID-19 Severities**

**
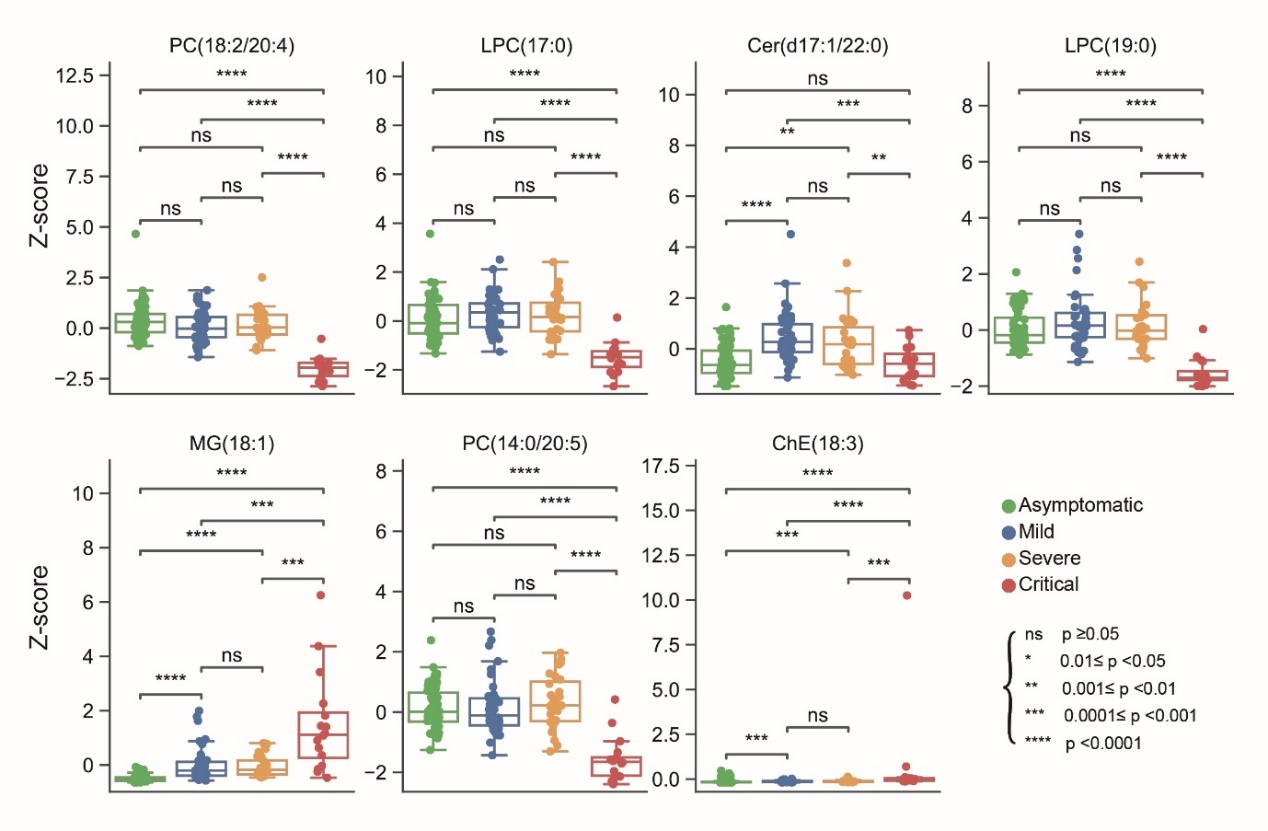
**

**Figure S4. Distribution of Lipids from the Top 60 Important Multi-omics Features among the 4 Types of COVID-19 Severities**

**
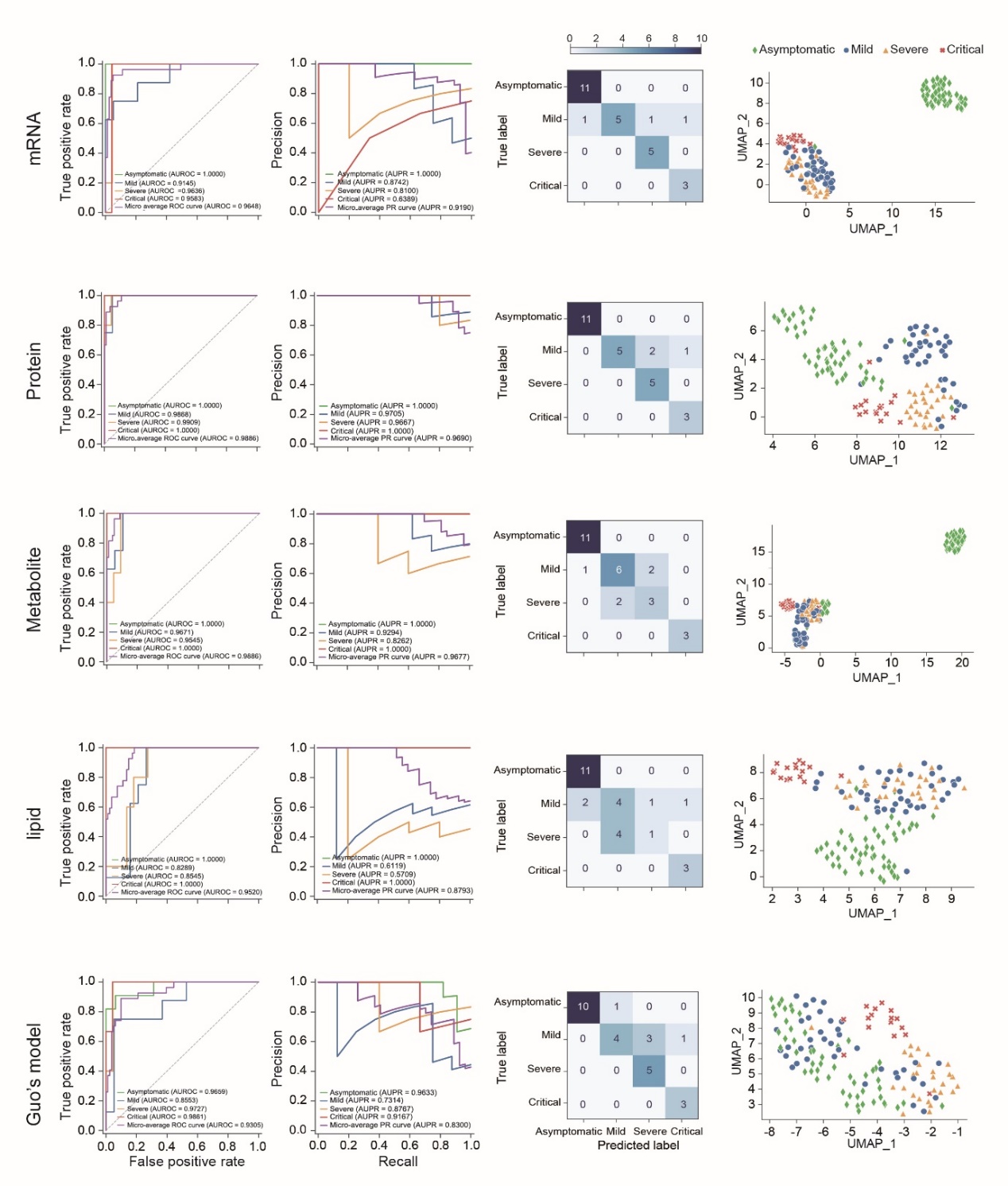
**

**Figure S5. Prediction Performance of Machine Learning Models for Each Single-omics Data and Guo’s Method**

The performance was measured respectively in terms of ROC curve with micro-average AUROC (first column), PR curve with micro-average AUPR (second column), confusion matrix (third column), and UMAP plot (fourth column).


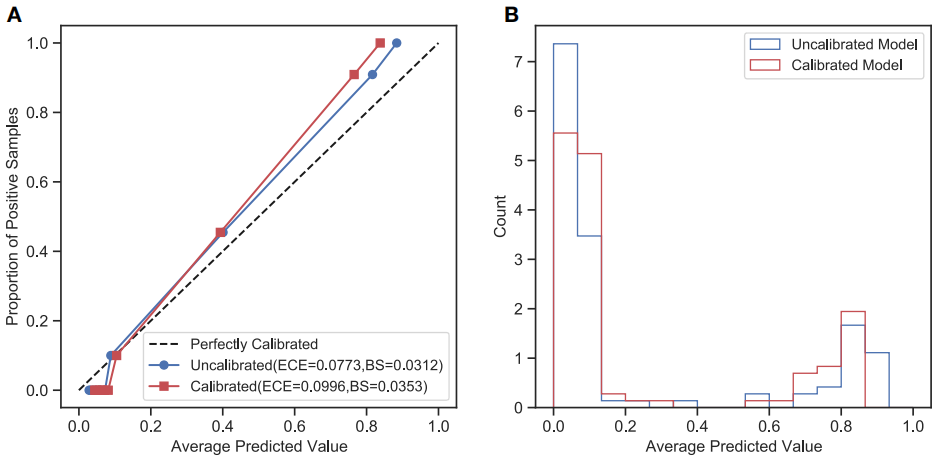


**Figure S6 A, Reliability diagrams on the test dataset for uncalibrated model and calibrated model by Platt scaling method with expected calibrator error (ECE) and brier score (BS). B, Histograms of the predicted values obtained from uncalibrated model and calibrated model.**
